# Taxonomic identification bias does not drive patterns of abundance and diversity in theropod dinosaurs

**DOI:** 10.1098/rsbl.2021.0168

**Published:** 2021-07-14

**Authors:** Daniel D. Cashmore, Richard J. Butler, Susannah C. R. Maidment

**Affiliations:** ^1^ School of Geography, Earth and Environmental Sciences, University of Birmingham, Edgbaston, Birmingham B15 2TT, UK; ^2^ Department of Earth Sciences, Natural History Museum, Cromwell Road, London SW7 5BD, UK

**Keywords:** taxonomic identification, bias, theropod dinosaurs, diagnostic, preservation

## Abstract

The ability of palaeontologists to correctly diagnose and classify new fossil species from incomplete morphological data is fundamental to our understanding of evolution. Different parts of the vertebrate skeleton have different likelihoods of fossil preservation and varying amounts of taxonomic information, which could bias our interpretations of fossil material. Substantial previous research has focused on the diversity and macroevolution of non-avian theropod dinosaurs. Theropods provide a rich dataset for analysis of the interactions between taxonomic diagnosability and fossil preservation. We use specimen data and formal taxonomic diagnoses to create a new metric, the Likelihood of Diagnosis, which quantifies the diagnostic likelihood of fossil species in relation to bone preservation potential. We use this to assess whether a taxonomic identification bias impacts the non-avian theropod fossil record. We find that the patterns of differential species abundance and clade diversity are not a consequence of their relative diagnosability. Although there are other factors that bias the theropod fossil record that are not investigated here, our results suggest that patterns of relative abundance and diversity for theropods might be more representative of Mesozoic ecology than often considered.

## 1. Introduction

In order to understand past ecology and key evolutionary changes, palaeontologists must be able to correctly estimate relative or absolute species abundance and diversity [1]. The imperfection of the fossil record means spatial, temporal and sampling biases [2–9] likely limit our knowledge, with many recent studies attempting to understand connections between apparent biological patterns and biases [10–17], and to quantify the level of ‘missing’ information in the fossil record [18–26]. However, a critical but less examined factor influencing interpretations is our ability to correctly identify fossil species [27–33]. In the tetrapod fossil record, inconsistent fossilization not only occurs on large spatial and temporal scales, but also across the individual bones of the skeleton. Furthermore, unique characters diagnosing species or wider clades (autapomorphies and synapomorphies) are also differentially distributed across the skeleton depending on the individual species or taxonomic group. Therefore, if the diagnostic characters of a particular taxonomic group are present on bones that are commonly preserved in the fossil record, palaeontologists should be able to more readily identify those fossils and distinguish species. The variable likelihood of preservation of individual bones and the variable distribution of taxonomically informative characters across the skeleton could therefore play pivotal roles in estimates of past abundance and diversity.

Here, we investigate whether a taxonomic identification bias is present in non-avian theropod dinosaurs. Theropods are one of the most intensively studied groups of fossil vertebrates [34–36], with a substantial interest in their macroevolutionary patterns [2,6,8,11–13,15,17,37–44], and they provide a rich source of data to explore connections between fossil preservation and taxonomic diagnosability. Owing to the abundant identification of fossils of some individual species, for example, *Allosaurus fragilis* in the Morrison Formation (e.g. [45,46]), we hypothesize that certain theropod subgroups are ‘easier’ to identify than others owing to fortunate combinations of bone preservation potential and distribution of diagnostic characters across the skeleton, leading to higher quantities of discoveries. We exclusively test for this potential bias on a global scale by quantifying the diagnostic quality of the fossil material of each theropod species and statistically comparing this to estimates of abundance at different taxonomic and spatio-temporal scales.

## 2. Material and methods

### (a) Likelihood of Diagnosis metric

**Figure 1.**
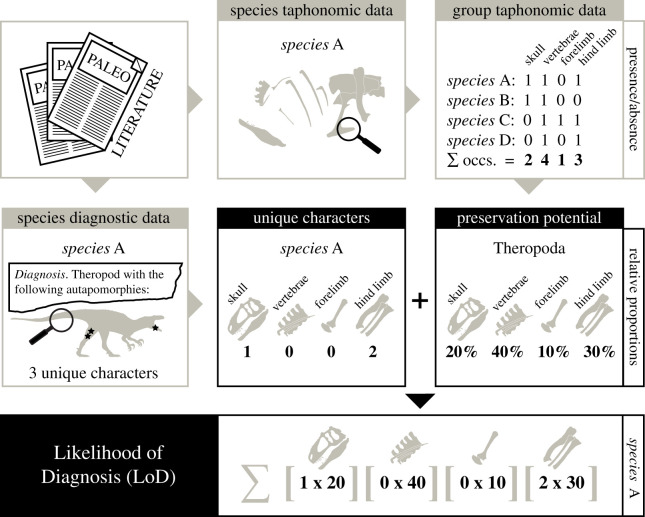
A diagrammatical representation of the process and methodology behind the Likelihood of Diagnosis (LoD) metric.

We updated (August 2020) an existing skeletal completeness dataset [25] to obtain the presence/absence data for each individual skeletal element (elements occurring within series (e.g. teeth, vertebrae, ribs and digits) were treated as one individual element; see electronic supplementary material) of all published non-avian theropod species (except those known only from isolated teeth [32]) and 69 unnamed, but phylogenetically informative, specimens previously included in cladistic analyses [47]. The total number of occurrences of each skeletal element was then calculated from all theropod specimens, and the proportion constituted by each element relative to all known theropod elements was used as its ‘global’ preservation potential (figure 1; see electronic supplementary material, ‘Supplementary methodology’ for data limitations).

Taxonomic diagnoses within the published literature define the most distinguishing features of fossil species in an easily accessible format. From these diagnostic summaries, we gathered the number of autapomorphies identified for each skeletal element for all valid theropod species (see ‘Theropod Diagnoses data’ [47]) (figure 1). All plesiomorphic, synapomorphic and differential diagnostic references to individual elements were ignored, but ‘unique combinations’ of characters were included. The total ‘unique combination’ of characters was regarded as equivalent to a single autapomorphy. Therefore, for each species with such diagnoses, the individual characters were scored as a proportion of the sum of all the characters (i.e. for a ‘unique combination’ of four characters, each character represents 25% of an autapomorphy) [47]. We incorporated diagnoses from formal systematic palaeontology sections, and only included data from post-1980 diagnoses, because, generally, before this time autapomorphies were not explicitly defined in diagnoses. Unique characters referring to entire body partitions (e.g. skull length), integument, fenestrae with contacts with multiple elements or the association of multiple elements (e.g. measurement ratios between two bones) could not be assigned to specific elements and were therefore excluded (see electronic supplementary material, ‘Supplementary methodology’ for data limitations).

For a given species, the number of unique characters (Ch) assigned to each skeletal element was multiplied by the ‘global’ preservation potential (PP) of each element, and the resulting scores were summed to produce a Likelihood of Diagnosis (LoD) score for that species (figure 1) (LoD = ∑[Ch × PP] + …*n*). A high LoD means that a higher number of unique characters have been identified for a species and/or that identified autapomorphies are distributed on skeletal elements that are commonly preserved.

**Figure 2.**
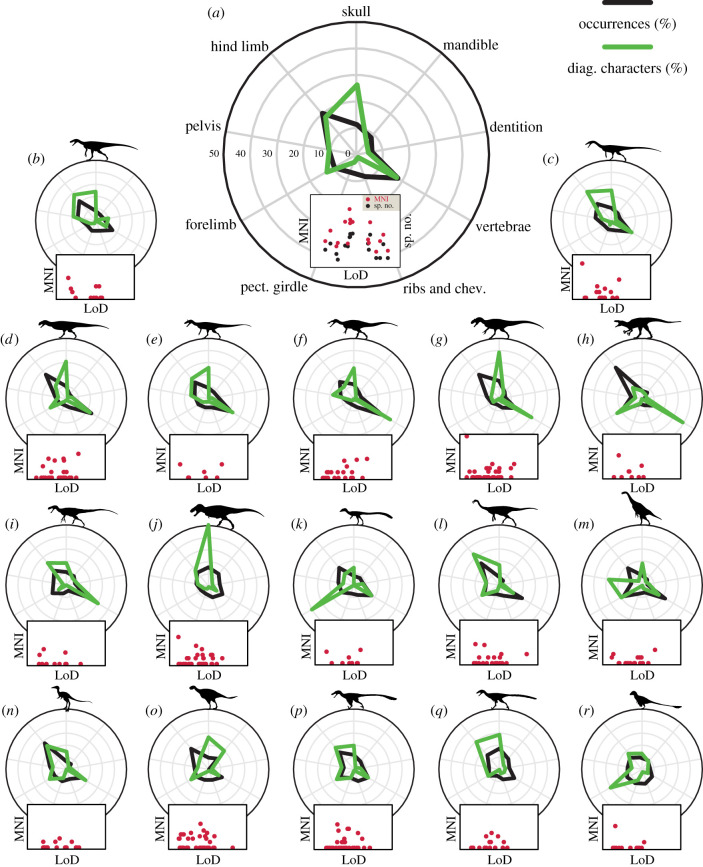
Radial plots depicting the relative percentage of occurrences of, and unique diagnostic characters assigned to, the major skeletal regions of Theropoda. All subgroup outer circles represent 50%. Scatterplots depict relationships between the LoD and species richness (black) and minimum number of individuals (MNI) (red) for summarized subgroup data (*a*) and between LoD and MNI per species (*b–r*). (*a*) All theropod species; (*b*) ‘basal’ Theropoda; (*c*) basal Neotheropoda; (*d*) Ceratosauria; (*e*) basal Tetanurae; (*f*) Megalosauroidea; (*g*) Allosauroidea; (*h*) Megaraptora; (*i*) basal Coelurosauria; (*j*) Tyrannosauroidea; (*k*) Compsognathidae; (*l*) Ornithomimosauria; (*m*) Therizinosauria; (*n*) Alvarezsauroidea; (*o*) Oviraptorosauria; (*p*) Dromaeosauridae; (*q*) Troodontidae; (*r*) non-avialan Paraves. Abbreviations: chev., chevrons; pect., pectoral. Silhouettes used include work by S. Hartman, T Michael Keesey, T. Tischler, J. Conway, Funkmonk and M. Martyniuk (http://phylopic.org/; CC BY-SA 3.0).

To evaluate the likelihood of diagnosing all of the known species in a more inclusive grouping of data (i.e. taxonomic subgroup, geological formation, time bin), we calculated the mean LoD scores from constituent species. To ensure data were approximately normally distributed (electronic supplementary material, figures S1–S3) [47] and mean values not skewed by outliers, we logged the LoD scores prior to mean calculation. Species were split into major theropod subgroups following phylogenetic relationships used in Cashmore and Butler [25] (figure 2; see electronic supplementary material, ‘theropod relationships'). For each subgroup, we further calculated the relative proportion of unique characters and relative proportion of skeletal element occurrences (figure 2; electronic supplementary material, table S1).

### (b) Abundance proxies

We calculated the minimum number of individuals (MNI) for each theropod species based on the number of duplicated elements associated with each species in our skeletal completeness dataset [47], cross-referenced with published literature. MNIs for taxonomic, geological formation and temporal groupings were summed from all known species (including species lacking ‘autapomorphic’ diagnoses) and indeterminate specimens, and ratios of MNI to valid species richness were also used as an abundance proxy [47].

We further calculated a number of abundance proxies from theropod data in the Paleobiology Database (PBDB) (https://paleobiodb.org) (downloaded on 07/07/20). For each valid species, we calculated the number of occurrences, individuals, and the ratio of individuals to unique localities (i.e. collections) [47]. The same proxies were calculated for each taxonomic subgroup, formation and time bin, but these also included specimens only identified to higher taxonomic levels (e.g. Tyrannosauridae indet.) [47]. Additionally, for each grouping, we calculated the ratio of individuals per species, and species and individuals per locality, as other potentially informative abundance proxies.

### (c) Statistical tests

For each species, we statistically compared the LoD scores with their MNI, across all theropods, and within each subgroup, Mesozoic stage, and within each of the five most species-rich geological formations. The species-level PBDB abundance proxies were solely compared across all Theropoda. Across all taxonomic subgroups, formations and time bins, mean logged LoD scores were statistically compared to species richness, summed MNI and PBDB subgroup abundance proxies. Stage-level time bins were chosen as most Mesozoic stratigraphic data are not well constrained to finer scales. Minimum and maximum stage dates were determined from Walker *et al*. [48]. Species that were present over multiple geological stages, or have an uncertain stratigraphic age, were included in each stage in which they potentially were present.

Generalized least-squares regression (GLS) was used for linear comparisons, implemented using the function gls() in the R package nlme [49]. A first-order autoregressive model (corARMA) was applied to temporal data to reduce the chances of overestimating statistical significance owing to autocorrelation. Prior to analysis, log-transformation was applied to ensure normality of residuals and homoscedasticity (constant variance). We further calculated a likelihood-ratio based pseudo-*R*^2^ value by using the function r.squaredLR() of the R package MuMIn [50].

R (v. 3.1) [51] was used to perform all statistical tests and initially create all plots. Radial plots were created with the package plotrix [52].

## 3. Results

Theropod skeletal regions with the highest preservation potential are the hind limb and vertebrae, with the caudal vertebrae, tibia, femur and metatarsals preserved most frequently. Most theropod diagnostic characters come from the skull, hind limb and vertebrae, with the maxilla, metatarsals and cervical and caudal vertebrae the predominant contributors (figure 2*a*; electronic supplementary material, table S1).

We find no significant relationship between species LoD and MNI or any PBDB abundance proxy across all theropods (table 1), within each subgroup (figure 2*b–r*), each relevant geological formation and time bin (electronic supplementary material, tables S2–S4). Compsognathidae and Ornithomimosauria have the highest mean LoD scores of all subgroups, while non-avian Paraves and Megalosauroidea have the lowest [47]. Temporal fluctuations in mean LoD are limited [47], but there is a very gentle rise through time after an initial outlying peak in the Carnian. There are no significant relationships between mean LoD and species richness, MNI or any PBDB abundance proxy across each subgroup (figure 2*a* and table 1), across each formation or through geological time (table 2).

**Table 1.** Results of comparisons between LoD and select abundance proxies at different taxonomic scales using generalized least-squares regression (GLS).

comparison	slope	*t*-value	*R*^2^	*p*-value
species LoD ∼ MNI	0.0508	0.85	0.0020	0.40
species LoD ∼ PBDB individuals	0.0010	0.02	0.0000	0.99
species LoD ∼ PBDB occurrences	−0.0220	−0.33	0.0003	0.74
species LoD ∼ PBDB individuals per locality	0.0639	0.43	0.0006	0.67
subgroup LoD ∼ MNI	−0.0507	−0.94	0.0557	0.36
subgroup LoD ∼ species richness	−0.0534	−0.85	0.0461	0.41
subgroup LoD ∼ MNI per species	−0.0413	−0.38	0.0097	0.71
subgroup LoD ∼ PBDB individuals	−0.0157	−0.53	0.0181	0.61
subgroup LoD ∼ PBDB species richness	−0.0453	−0.73	0.0345	0.48
subgroup LoD ∼ PBDB individuals per species	−0.0118	−0.26	0.0046	0.80
subgroup LoD ∼ PBDB occurrences	−0.0152	−0.53	0.0185	0.60
subgroup LoD ∼ PBDB individuals per locality	−0.0601	−1.05	0.0679	0.31
subgroup LoD ∼ PBDB species per locality	−0.0531	−0.78	0.0393	0.45

**Table 2.** Results of comparisons between LoD and species richness and select abundance proxies at different spatio-temporal scales using GLS.

comparison	slope	*t*-value	*R*^2^	*p*-value
formation LoD ∼ MNI	0.0143	0.27	0.0005	0.79
formation LoD ∼ species richness	−0.0584	−0.76	0.0036	0.45
formation LoD ∼ MNI per species	0.0830	1.38	0.0116	0.17
formation LoD ∼ PBDB individuals	−0.0134	−0.33	0.0007	0.74
formation LoD ∼ PBDB species richness	−0.0482	−0.68	0.0031	0.50
formation LoD ∼ PBDB individuals per species	−0.0513	−0.84	0.0047	0.40
formation LoD ∼ PBDB occurrences	−0.0155	−0.38	0.0009	0.71
formation LoD ∼ PBDB individuals per locality	−0.1749	−1.45	0.0132	0.15
formation LoD ∼ PBDB species per locality	−0.0090	−0.12	0.0001	0.90
time LoD ∼ MNI	0.0459	1.60	0.0841	0.12
time LoD ∼ species richness	0.0466	1.51	0.0689	0.14
time LoD ∼ MNI per species	0.0317	0.54	0.0120	0.59
time LoD ∼ PBDB individuals	0.0334	1.41	0.0482	0.17
time LoD ∼ PBDB species richness	0.0438	1.37	0.0541	0.18
time LoD ∼ PBDB individuals per species	0.0144	0.25	0.0019	0.80
time LoD ∼ PBDB occurrences	0.0372	1.69	0.0679	0.10
time LoD ∼ PBDB individuals per locality	0.0179	0.10	0.0006	0.92
time LoD ∼ PBDB species per locality	−0.0152	−0.19	0.0015	0.85

## 4. Discussion

Our results suggest different theropod species and subgroups do have different chances of being correctly identified; however, statistical analyses suggest these differences have little impact on the relative abundance and diversity signals that we derive from the fossil record. Therefore, our understanding of the relative abundances of theropods within ecosystems, and the relative diversity of theropod subgroups to one another, may be better than pessimistic interpretations suggest [1,23,25,31]. This implies that these aspects of theropod diversity patterns outlined in many studies are at least moderately reliable for understanding theropod evolution. Nevertheless, various spatial and taphonomic factors still impact the theropod fossil record and perceived macroevolutionary signals. For example, specimens of Compsognathidae are almost entirely derived from localities of exceptional preservation, and as many of their diagnostic characters are attributed to the manus (figure 2; electronic supplementary material, table S1), which has only moderate preservation potential, it may therefore be relatively difficult to identify fragmentary material from other deposits.

Allosauroidea and Tyrannosauroidea have strikingly higher proportions of diagnostic skull characters in comparison to other subgroups (figure 2*g*,*j*) [53,54], the vast majority pertaining to the maxilla (electronic supplementary material, table S1). The identification of more diagnostic characters on that element may be a true biological signal reflecting strong cranial selection pressures [55–57], but could also be owing to variable worker interpretation and potential over-atomization of characters [34–36,53,55–56,58,59], which can have important implications for phylogenetic interpretations [53,57,60]. Conversely, the hind limbs of Megaraptora and Allosauroidea have high preservation potential but relatively few diagnostic characters and thus hind limb elements might be underused as a source of data for these groups [35] (figure 2). Character differences between subgroups could be related to a multitude of factors, including fossil preservation quality [25,26,35], bone size and robustness [23,31], geographic extent [34–36], author affiliations and potential clade study bias [34–36,53].

Oviraptorosaurs, dromaeosaurids, allosauroids and tyrannosauroids have both the highest species richness and MNI of all the subgroups. Notably, they also have the highest skeletal coverage of diagnostic characters (62–82%) (electronic supplementary material, table S1), possibly enabling more specimens and species to be identified from limited material that may otherwise be considered undiagnostic, potentially enabling stronger understanding of phylogenetic relationships [56,57].

We have defined LoD as a new metric quantifying the researcher ability to identify individual species. Within the LoD, the likelihood of recognizing new specimens of a species is effectively controlled by the number of unique characters assigned to it, which does not necessarily reflect the reality of identifications in the field or museum collections. For example, *A. fragilis* and *Tyrannosaurus rex* are two species known from a high abundance of material, yet both lack an up-to-date formal diagnosis [47], and therefore lack a quantifiable diagnosability score. In practice, additional specimens of these, and other apparently common species, are in many cases identified by general morphological similarity rather than specific autapomorphies. Therefore, LoD does not fully capture how new specimens are assigned to species and abundance proxies may be skewed by these ‘generalized’ identifications, potentially causing the lack of statistical relationship between LoD and abundance (tables 1 and 2). Furthermore, LoD is itself likely influenced by the variable preservation and sampling biases that impact the fossil record, but understanding this is beyond the scope of this study. Despite these limitations, we believe LoD is an efficient approach to quantify the diagnosability of fossil material and specifically address potential taxonomic identification bias.

Although the theropod fossil record is no doubt biased by various preservation and historical sampling factors, we cannot identify particular formations or time bins to which palaeontologists have applied a significantly different set of identification criteria, which biases diversity or abundance patterns. We therefore should have confidence in the manner in which workers gather taxonomic data, and probably more confidence in the ecological and evolutionary information derived from the theropod fossil record: higher relative abundance or diversity of a particular species or clade, or time bin, are not the result of identification bias, but could be owing to other known preservation biases, or actually represent real patterns. While taxonomic identification is likely not a major source of bias for theropod dinosaurs, this conclusion cannot be widely applied to the entire vertebrate fossil record.

## Data accessibility

Data and code available from the Dryad Digital Repository: https://doi.org/10.5061/dryad.x95x69phs [47].

The data are provided in the electronic supplementary material [61].
